# A Comparison of Serum Lipase Levels and Clinical Outcome Trends in Hospitalized Pediatric Patients With Acute Pancreatitis: A Single-Center Retrospective Study

**DOI:** 10.7759/cureus.103489

**Published:** 2026-02-12

**Authors:** Sandeep K Puri, Amreen Masthan, David J Freestone, Andrew S Huang-Pacheco, Ruben E Quiros-Tejeira

**Affiliations:** 1 Pediatric Gastroenterology, University of Nebraska Medical Center, Omaha, USA

**Keywords:** acute pancreatitis, healthcare cost, length of stay, lipase trending, quality improvement, retrospective study, serum lipase

## Abstract

Background

Serum lipase is widely used for the diagnosis of acute pancreatitis (AP) in pediatric patients; however, its prognostic value remains unclear. Despite limited evidence supporting serial monitoring, lipase levels are frequently trended during hospitalization. This study aimed to evaluate whether trending serum lipase levels are associated with improved clinical outcomes in children hospitalized with AP.

Methodology

We conducted a single-center retrospective chart review of pediatric patients (0-19 years) admitted with AP at Children’s Hospital & Medical Center between January 1, 2021, and December 31, 2022. Diagnosis was based on the INSPPIRE criteria. Patients were categorized into the following two groups: those who had serum lipase levels trended after diagnosis and those who did not. Clinical outcomes, including persistence of abdominal pain, nausea, and/or vomiting at 48 and 96 hours; length of stay (LOS); type of intravenous fluids administered; analgesic use; associated risk factors; and cost of laboratory testing, were analyzed.

Results

A total of 65 patients met the inclusion criteria. Of these, 77% had serial lipase levels measured. There was no statistically significant difference in symptom persistence at 48 hours (63.8% in trended vs. 78.6% in non-trended; p = 0.35) or at 96 hours (82.9% vs. 100%; p = 0.58). Patients in the trended group had a significantly longer median LOS (4.0 days) compared to the non-trended group (2.5 days) (p = 0.0129). The mean laboratory cost was higher in the trended group ($184.0) compared to the non-trended group ($66.0). No meaningful prognostic correlation was identified between serial lipase levels and clinical improvement.

Conclusions

Serial trending of serum lipase levels in hospitalized pediatric patients with AP does not provide prognostic value regarding symptom resolution and is associated with increased hospital LOS and higher healthcare costs. Lipase testing should be limited to diagnostic purposes rather than routine monitoring during hospitalization.

## Introduction

Acute pancreatitis (AP) is an increasingly recognized cause of hospitalizations in children and is associated with significant morbidity and healthcare utilization. The incidence of AP in children is rising. Etiologies include structural/anatomic, obstructive/biliary, trauma, infections, toxins, metabolic, systemic illnesses, inborn errors of metabolism, and genetic predispositions. The estimated incidence is approximately 1-3 cases per 10,000 children [1,2].

The diagnosis of pediatric AP is established using standardized criteria, most commonly the International Study Group of Pediatric Pancreatitis: In Search for a Cure (INSPPIRE) definition, which relies on a combination of characteristic abdominal pain, elevated pancreatic enzymes, and/or imaging findings. Once the diagnosis is made, management is largely supportive and guided by clinical status rather than biochemical normalization [3].

Serum lipase is a sensitive and specific marker for the diagnosis of AP and is routinely obtained at presentation. However, lipase levels may remain elevated for several days after symptom onset and do not reliably correlate with disease severity, clinical improvement, or resolution of symptoms. Despite this, repeat measurement of serum lipase after diagnosis remains common in hospitalized children, often driven by practice patterns rather than evidence. This practice may contribute to unnecessary laboratory testing, increased costs, and potential misalignment between laboratory trends and clinical decision‑making [2].

Existing adult and pediatric literature suggests that serial monitoring of pancreatic enzymes does not provide prognostic value once AP is diagnosed and should not guide clinical management. Nonetheless, data evaluating the association between lipase trending and symptom‑based clinical outcomes in pediatric AP remain limited, particularly in real‑world inpatient settings.

It is not uncommon for many institutions to trend serum lipase levels in hospitalized patients. Trending the serum lipase levels in hospitalized pediatric patients may put unnecessary financial and emotional burden on families, insurance companies, and institutions [4,5].

The primary objective of this study was to evaluate the association between serial serum lipase trending and short-term clinical outcomes in children hospitalized with AP. Clinical outcomes were defined as the presence of ongoing abdominal pain, nausea, and/or vomiting at 48 and 96 hours after admission. Secondary objectives included examining the association between lipase trending and hospital length of stay (LOS), opioid use, intravenous fluid type, and hospitalization costs. By focusing on clinically meaningful outcomes rather than biochemical normalization, this study aims to assess the utility of serial lipase testing in routine pediatric AP management.

## Materials and methods

We identified 65 inpatient encounters at Children’s Nebraska (CHMC) from January 1, 2021, to December 31, 2022. The inclusion criteria were (1) patients aged 0-19 years hospitalized with AP; (2) patients seen by the PI, SI, or Pediatric GI team at CHMC; and (3) patients seen by other inpatient services at CHMC. The exclusion criteria were (1) patients aged 20 years or older and (2) patients who did not meet the inclusion criteria. A total of 61 patients met the inclusion criteria. Clinical outcomes were defined as the presence of ongoing abdominal pain, nausea, and/or vomiting at 48 and 96 hours after admission.

Data collection

The data collected were serum lipase levels, frequency of serum lipase testing during the same inpatient encounter, imaging studies, if performed, risk factors, complications, and body mass index (BMI) (Centers for Disease Control and Prevention guidelines for children ≥2 years; weight-for-age percentile for children <2 years). We also reviewed and compared the type of fluids (lactated Ringer’s vs. non-lactated Ringer’s), choice of pain medications (opioids vs. non-opioids), and correlation between serum lipase levels and risk factors. For this study, lipase trending was defined as more than one serum lipase measurement obtained after diagnosis. Patients with only a single lipase measurement were classified as not trended. One patient with a single repeat measurement obtained within the diagnostic window was categorized as trended based on the clinical context.

Statistical methods

All categorical data were summarized in two-way tables with counts and proportions. As a few cell counts were relatively small, to test statistical independence, Fisher’s exact test was applied. Continuous predictors with binary outcomes were evaluated with logistic regression. Continuous data, such as cost and lipase levels, were right-skewed, and a square root transformation was applied to improve distributional properties. The transformed continuous outcomes were evaluated with either a t-test or one-way analysis of variance. LOS was evaluated using a Kaplan-Meier analysis, including a graphical display. Statistical analyses were generated with the FREQ, LOGISTIC, and NPAR1WAY procedures from SAS/STAT software. Graphs were produced with the SGPLOT procedure from SAS/Base software, Version 9.4 (© 2016) of the SAS System for Windows (Cary, NC). For all inferential analyses, the corresponding test statistic (t, χ², or log-rank χ²) and degrees of freedom were reported alongside p-values.

Given the retrospective design, analyses were exploratory and intended to assess associations rather than causation. No multivariable adjustment was performed, and potential confounding by indication is addressed qualitatively in the Discussion.

## Results

A total of 61 patient encounters met the study criteria. In 77% of patients (47/61), serum lipase level was trended. The number of patients available for outcome assessment decreased over time due to discharge before the 48- and 96-hour time points; therefore, analyses at each time point include only patients still hospitalized at that interval. Patient demographics and serum lipase measurement frequency are presented in Table 1.

**Table 1:** Patient demographics and serum lipase measurement frequency. IV: intravenous; LR: lactated Ringer’s

Patient demographics		n (%)
Ethnicity	Hispanic	9 (14.8)
	Non-Hispanic	52 (85.2)
Sex	Female	35 (57.4)
	Male	26 (42.6)
Lipase trended?	No	14 (23)
	Yes	47 (77)
Pain management	Non-narcotics	33 (54.1)
	Narcotics	28 (45.9)
Type of IV fluids	Non LR	25 (41)
	LR	36 (59)
Risk factors/Complications	Yes	47 (79.7)
	No	12 (20.3)
	Missing	2 (0.03)
Readmitted within 7 days?	No	57 (93.4)
	Yes	4 (6.6)

Association between serial serum lipase trending and short‑term clinical outcomes

To assess the association between serial serum lipase trending and clinical outcomes, categorical outcomes at 48 and 96 hours were compared between groups using Fisher’s exact test, given small cell counts. At both time points, there was no statistically significant association between lipase trending and the presence of ongoing abdominal pain, nausea, and/or vomiting.

Although a higher percentage of patients without lipase trending had persistent clinical symptoms at both 48 and 96 hours compared to those with lipase trending, these differences were not statistically significant, as reflected by the corresponding p-values. These findings indicate that serial lipase trending was not associated with improved short‑term clinical outcomes in this cohort (Tables 2-4).

**Table 2:** Association between lipase trending and clinical outcomes at 48 hours. This table compares the presence of clinical symptoms at 48 hours between patients with trended and non‑trended serum lipase levels.

Clinical outcomes	Lipase trended	Lipase not trended	χ² (df)	P-value
Present, n (%)	30 (63.8)	11 (78.6)	0.87 (1)	0.35
Absent, n (%)	17 (36.2)	3 (21.4)		

**Table 3:** Association between lipase trending and clinical outcomes at 96 hours. This table compares the presence of clinical symptoms at 96 hours between patients with trended and non‑trended serum lipase levels.

Clinical outcomes	Lipase trended	Lipase not trended	χ² (df)	P-value
Yes, n (%)	29 (82.9)	5(100)	0.30 (1)	0.58
No, n (%)	6 (17.1)	0 (0)		

**Table 4:** Association between lipase trending and clinical outcomes at greater than 96 hours. This table compares the presence of clinical symptoms at greater than 96 hours between patients with trended and non‑trended serum lipase levels.

Clinical outcomes	Lipase trended	Lipase not trended	χ² (df)	P-value
Yes, n (%)	25 (92.6)	4 (100)	0.12 (1)	1.00
No, n (%)	2 (7.4)	0 (0)		

Association between serum lipase levels and short‑term clinical outcomes

Due to marked right skewness in serum lipase values, a square‑root transformation was applied before analysis to reduce the influence of extreme values. In the model evaluating clinical outcomes at 48 hours, the parameter estimate for transformed lipase level was negative, corresponding to an odds ratio of less than 1. This indicates a directional association in which higher lipase values were associated with a lower probability of persistent clinical symptoms; however, this association was not statistically significant (p = 0.397).

Model discrimination was assessed using the area under the receiver operating characteristic curve (AUC). The AUC was 0.539, indicating poor discriminatory ability and suggesting that serum lipase level alone had limited value in distinguishing between patients with and without persistent clinical outcomes in this cohort (Table 5).

**Table 5:** Clinical outcomes at 48 hours by square root-transformed serum lipase level.

Clinical outcome	n	Median SQRT lipase level	Mean SQRT lipase level	Minimum SQRT lipase level	Maximum SQRT lipase level
Present	40	63.2	63.5	4.1	173.2
Absent	20	62.4	72.2	6.2	158.1

At 96 hours, the interpretation was similar to that at 48 hours. The positive coefficient (0.0118) and an odds ratio greater than 1 (1.012) suggested a trend toward increased probability; however, this was not statistically significant (p = 0.352) (Table 6).

**Table 6:** Clinical outcomes at 96 hours by square root-transformed serum lipase level.

Clinical outcome	n	Median SQRT lipase level	Mean SQRT lipase level	Minimum SQRT lipase level	Maximum SQRT lipase level
Present	33	63.2	73.1	6.2	173.2
Absent	6	54.3	55.7	27.5	83.7

For the results >96 hours, interpretation was not performed due to the extremely small number of “absent” responses (n = 2) (Table 7).

**Table 7:** Clinical outcomes at greater than 96 hours versus SQRT lipase level(s).

Clinical outcome	n	Median SQRT lipase level	Mean SQRT lipase level	Minimum SQRT lipase level	Maximum SQRT lipase level
Present	29	63.2	74.5	6.2	173.2
Absent	2	87	87	53.9	120.1

Association between intravenous fluid type and short‑term clinical outcomes

Clinical outcomes at 48 and 96 hours were compared between patients receiving lactated Ringer’s and non-lactated Ringer’s intravenous fluids using Fisher’s exact test, given the presence of small cell counts in both tables. At both time points, the exact test p-values were relatively small, indicating a statistical association between fluid type and the presence of clinical outcomes.

At 48 hours, clinical outcomes were present in 77.8% (28/36) of patients who received lactated Ringer’s fluids compared with 52.0% (13/25) of those who received non-lactated Ringer’s fluids. Similarly, at 96 hours, clinical outcomes were present in 95.8% (23/24) of patients receiving lactated Ringer’s fluids compared with 68.8% (11/16) of those receiving non-lactated Ringer’s fluids.

These findings demonstrate an association between intravenous fluid type and short‑term clinical outcomes; however, given the retrospective design, small sample size, and potential confounding by indication, these results should be interpreted cautiously and not as evidence of a causal relationship (Tables 8-10).

**Table 8:** Association between intravenous fluid type and clinical outcomes at 48 hours. IV: intravenous; LR: lactated Ringer’s

Clinical outcome at 48 hours	LR IV fluids	Non-LR IV fluids	χ² (df)	P-value
Present, n (%)	28 (77.8)	13 (52)	3.78 (1)	0.052
Absent, n (%)	8 (22.2)	12 (48)		

**Table 9:** Association between type of intravenous fluid and clinical outcome at 96 hours. IV: intravenous; LR: lactated Ringer’s

Clinical outcome at 96 hours	LR IV fluids	Non-LR IV fluids	χ² (df)	P-value
Present, n (%)	23 (95.8)	11 (68.8)	4.78 (1)	0.029
Absent, n (%)	1 (4.2)	5 (31.3)		

**Table 10:** Association between type of intravenous fluid and clinical outcome at greater than 96 hours. IV: intravenous; LR: lactated Ringer’s

Clinical outcome at > 96 h	LR IV fluids	Non LR IV fluids	χ² (df)	P-value
Present, n (%)	17 (100)	12 (85.7)	1.67	0.20
Absent, n (%)	0 (0)	2 (14.3)		

Association between opioid use and short‑term clinical outcomes

Clinical outcomes at 48 and 96 hours were compared between patients receiving opioid versus non‑opioid analgesia using Fisher’s exact test, given small cell counts in both tables. At both time points, the exact test p-values were relatively large, indicating no statistically significant differences in clinical outcomes between the two analgesic groups.

At 48 hours, clinical outcomes were present in 57.1% (16/28) of patients who received opioids compared with 75.8% (25/33) of those who received non‑opioid analgesia. Although a similar pattern was observed at 96 hours, these differences did not reach statistical significance. Overall, opioid use was not statistically associated with short‑term clinical outcomes in this cohort (Tables 11-13).

**Table 11:** Association between opioid use and clinical outcomes at 48 hours.

Outcomes at 48 hours	Narcotic pain management	Non-narcotic pain management	χ² (df)	P-value
Present, n (%)	16 (57.1)	25 (75.8)	2.38 (1)	0.17
Absent, n (%)	12 (42.9)	8 (24.2)		

**Table 12:** Association between opioid use and clinical outcomes at 96 hours.

Outcomes at 96 hours	Narcotic pain management	Non-narcotic pain management	χ² (df)	P-value
Present, n (%)	18 (85.7)	16 (84.2)	0.01 (1)	1.00
Absent, n (%)	3 (14.3)	3 (15.8)		

**Table 13:** Association between opioid use and clinical outcomes at greater than 96 hours.

Outcomes at > 96 hours	Narcotic pain management	Non-narcotic pain management	χ² (df)	P-value
Present, n (%)	16 (88.9)	13 (100)	1.33 (1)	0.50
Absent, n (%)	2 (11.1)	0 (0)		

Healthcare cost implications of guideline‑based management without serial serum lipase measurements

**Figure 1:**
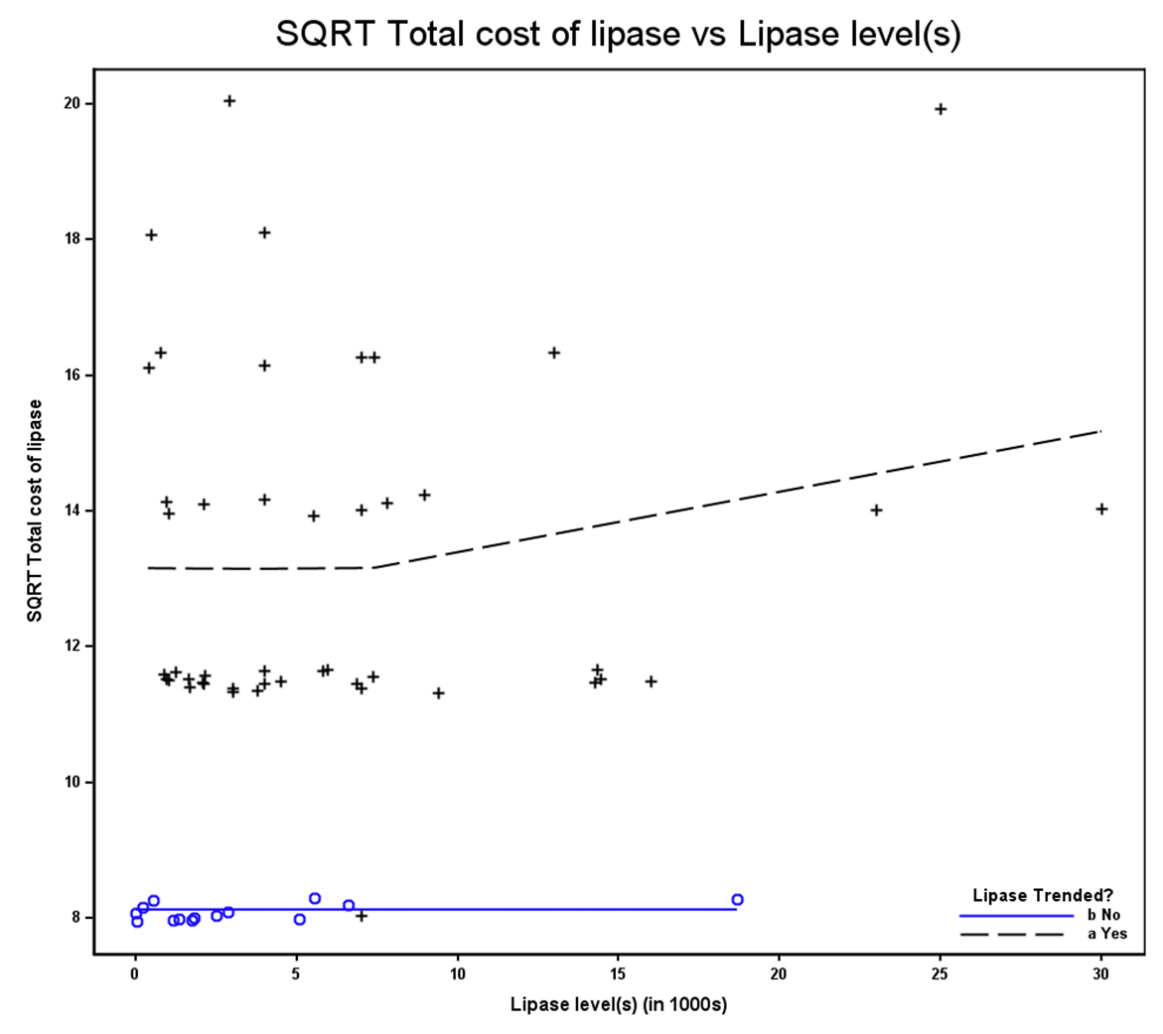
Hospitalization costs between groups.

Figure 1 compares hospitalization costs between groups. In the figure, the solid blue line represents patients in whom serum lipase was not trended (n = 14), corresponding to a constant cost of $66, plotted on the square‑root scale (√66 ≈ 8.1). The dashed line represents the estimated trend in cost among patients whose serum lipase levels were trended (n = 47). Beyond a lipase level of 100 U/L (√lipase = 10), the curve demonstrates an upward pattern; however, this segment is based on a small number of observations and should be interpreted cautiously, as it may not reflect a true increase in cost with higher lipase levels.

Because the cost data were right‑skewed, comparisons were performed on the square root-transformed cost scale. The group in which serum lipase was not trended exhibited no variability in cost (all values = $66), necessitating the use of an unequal‑variance model with variance for this group constrained near zero. Under these conditions, a conventional test statistic and corresponding p-value could not be calculated; therefore, the comparison is descriptive and reported as a mean difference on the transformed scale. Estimated means were subsequently back‑transformed to the original cost scale, and Table 14 presents the mean costs for the two lipase management groups.

**Table 14:** Mean laboratory cost by serum lipase trending status.

Lipase trended?	Mean
Yes	182.4
No	66
Difference	-116.4

In this sample, the estimated cost savings for the 47 patients who underwent serum lipase trending were calculated as $116.4 × 47 = $5,471. Because the non‑trended group exhibited no variability in cost (all values = $66), a formal test statistic and corresponding p-value could not be calculated; accordingly, all comparisons are descriptive.

The mean cost among the 47 patients with serum lipase trending was therefore descriptively compared with $66, representing the cost that would have been incurred had only a single serum lipase measurement been obtained. Mean costs are summarized in Table 15, along with the corresponding estimated cost savings under a single‑test strategy.

**Table 15:** Mean laboratory cost per patient by serum lipase trending status.

Lipase trended?	n	Mean
Yes	47	184.0
No	14	66.0

Based on the means of the data (not using a statistical model), the difference in the means is 118. Total cost savings is the difference, 118 x 47 = $5,546.

Association between serum lipase levels and the presence of one or more risk factors

The p-values in both the t-test and the Wilcoxon test are large (>0.85), indicating the means/medians are not different from each other (Table 16).

**Table 16:** Association between serum lipase levels and acute pancreatitis risk factors. Data are presented as mean ± SD or median (interquartile range), as appropriate. Group comparisons were performed using a two-sample t-test and a Wilcoxon rank-sum test. The t-test yielded t = −0.13 with 56 degrees of freedom (p = 0.90). Statistical significance was defined as p < 0.05. AP: acute pancreatitis

t-test summary for risk factors for AP	n	Nms	Median	Mean difference	Standard error	Lower limit	Upper limit	t	df	P-value (t-test)	P-value (Wilcoxon)
No risk	12	0	59	63.53	7.54	46.94	80.12	-0.13	56	0.9	0.85
Yes	46	1	62.4	65.04	5.75	53.45	76.62				
Difference (row 1–row 2)	–	–	–	-1.51	11.94	-25.43	22.41				

Gallstones were the most prevalent AP risk factor in the cohort. Both the two-sample t-test and the Wilcoxon rank-sum test demonstrated a statistically significant difference in square root-transformed serum lipase levels between patients with and without gallstones. The t-test yielded t = −3.62 with 44 degrees of freedom (p < 0.001), with concordant results from the Wilcoxon rank-sum test (p = 0.004).

Association between serum lipase trending and identified risk factors

Table 17 presents the association between serum lipase trending and risk factors. There was no statistical difference with a p-value of 0.45.

**Table 17:** Association between serum lipase trending and risk factors. AP: acute pancreatitis

Risk factors for AP	Lipase trended, n (%)	Lipase not trended, n (%)	χ² (df)	P-value
Yes	37 (78.7)	10 (21.3)	0.57 (1)	0.45
No	8 (66.7)	4 (33.3)		

Table 18 presents the largest frequencies of risk factors and complications. Frequencies of these items are too small to consider. Table 19 presents the association between gallstones and serum lipase trending.

**Table 18:** Largest frequencies of risk factors and complications for these factors. Data are presented as frequency. IBD: inflammatory bowel disease; DM: diabetes mellitus

Complications	Frequency
Gallstones	9
IBD	4
DM	5
Biliary cyst	3
Multiple	2
Multiple comorbidities	2

**Table 19:** Association between gallstones and serum lipase trending.

Gallstone	Lipase trended, n (%)	Lipase not trended, n (%)	χ² (df)	P-value
Yes	7 (77.8)	2 (22.2)	0.57 (1)	1.00
No	29 (78.4)	8 (21.6)		

Association between hospital length of stay and frequency of serum lipase testing

**Figure 2:**
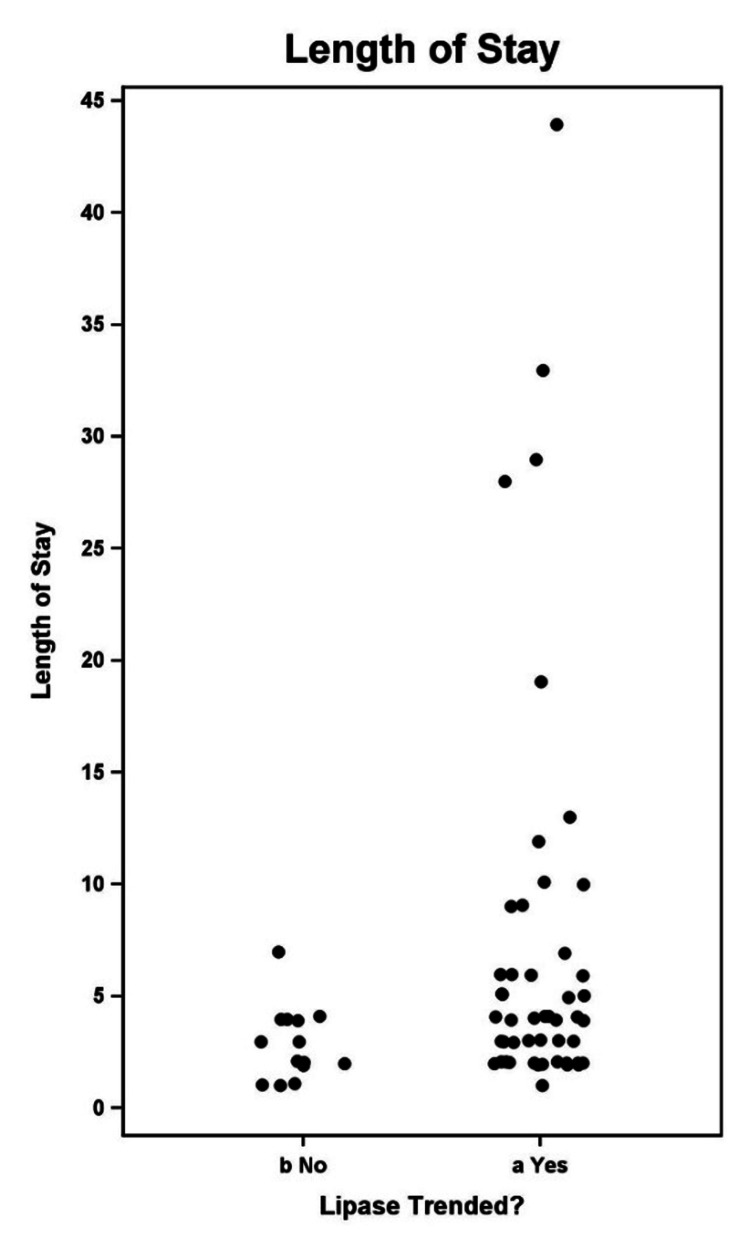
Hospital length of stay by lipase trending status.

Figure 2 illustrates the hospital LOS by lipase trending status.

Length of hospital stay was longer in the lipase‑trended group, with a median of 4.0 days compared with 2.5 days and a mean of 6.09 days compared with 2.86 days. Due to the absence of analyzable individual‑level time‑to‑event data, no formal statistical hypothesis testing was performed (Table 20). Table 21 presents a comparison between the two groups.

**Table 20:** Analysis variable: length of hospital stay. Length of hospital stay was longer in the lipase‑trended group, with a median of 4.0 days compared with 2.5 days and a mean of 6.09 days compared with 2.86 days. Due to the absence of analyzable individual‑level time‑to‑event data, no formal statistical hypothesis testing was performed.

Lipase trended	N	Median	Mean
No	14	2.50	2.86
Yes	47	4.00	6.09

**Table 21:** Comparison between two groups. Statistical significance was defined as p < 0.05.

Test	χ² (df)	P-value
Log-rank	6.1771 (1)	0.0129

**Figure 3:**
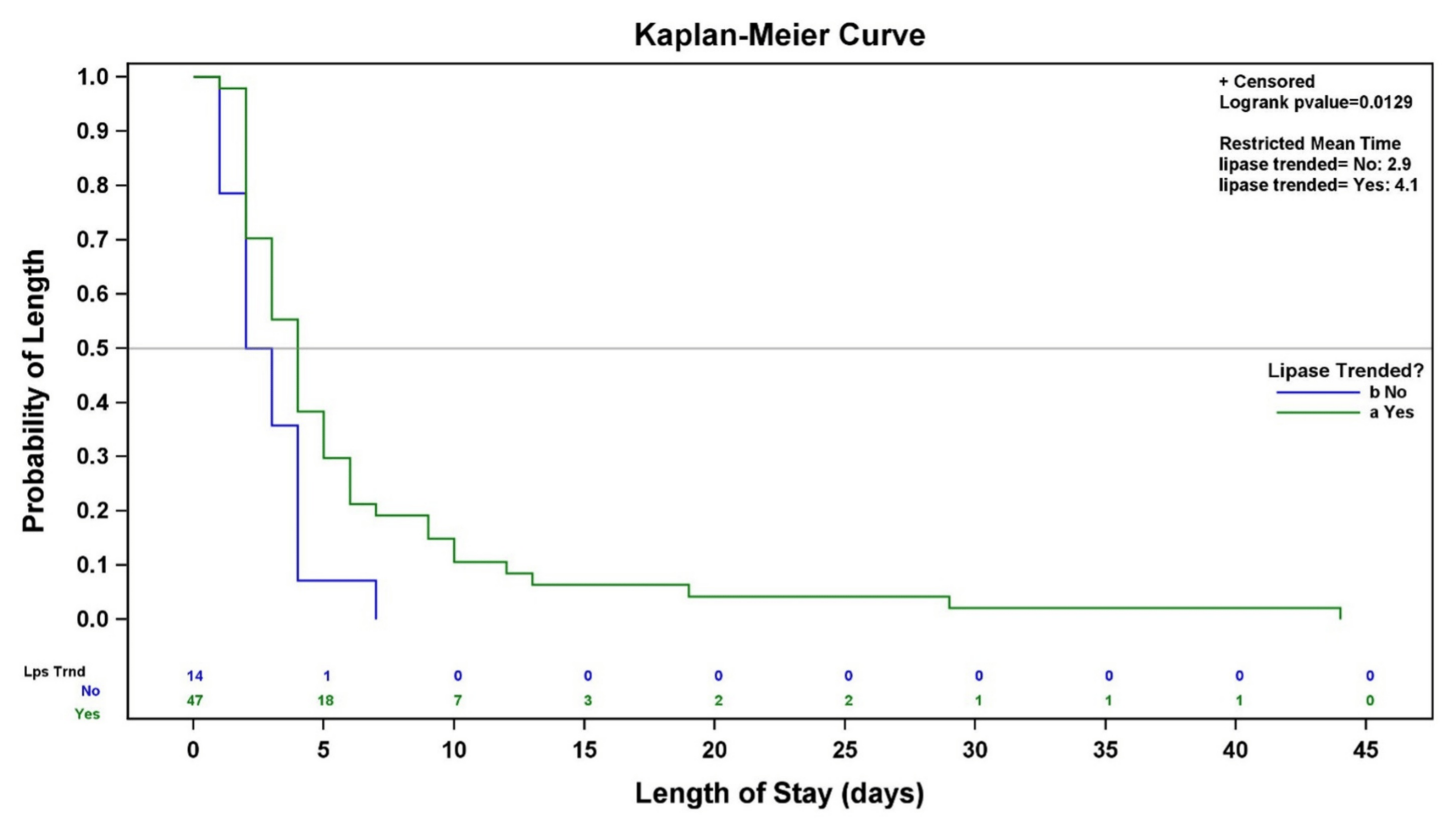
Kaplan–Meier curve of hospital length of stay by serum lipase trending. Kaplan–Meier curves depict the probability of remaining hospitalized over time. Censoring is indicated by tick marks. Group comparisons were performed using the log‑rank test (χ² = 6.18, df = 1, p = 0.0129), demonstrating a statistically significant difference between groups. Restricted mean survival time (RMST) is also shown (lipase not trended: 2.9 days; lipase trended: 4.1 days). Statistical significance was defined as p < 0.05.

Restricted mean survival time (RMST) estimates the average LOS within a specified follow‑up period using survival analysis methods and is related to, but distinct from, the median LOS. The RMST for patients with lipase trending was notably lower than the arithmetic mean LOS reported previously. This difference reflects the methodological distinction between RMST and the standard mean and is expected in this context, as LOS values are right‑skewed with a small number of prolonged hospitalizations, as illustrated in Figure 3.

## Discussion

This study evaluated the clinical utility of serial serum lipase measurement in hospitalized pediatric patients with AP. Consistent with existing literature, routine lipase trending after diagnosis was not associated with improvement in short‑term clinical outcomes [4].

Serum lipase elevation reflects pancreatic injury and is one of three diagnostic criteria for AP, along with characteristic abdominal pain and supportive imaging findings. Although lipase is often preferred over amylase because of greater specificity and, in some cases, diagnostic utility compared with imaging modalities, its role is primarily diagnostic. Lipase is present in several non‑pancreatic tissues [6] but is markedly more concentrated in pancreatic tissue [7]. Due to renal tubular reabsorption, serum lipase levels may remain elevated for prolonged periods, even after clinical recovery, limiting the usefulness of serial measurements in monitoring disease activity or severity [7].

In this retrospective cohort, serial lipase trending was not associated with symptom resolution at 48 or 96 hours. Although patients who underwent lipase trending had a longer hospital LOS, this finding represents an association rather than a causal relationship and is likely influenced by confounding by indication. Patients with more severe or persistent symptoms may have been more likely to undergo repeat testing and prolonged hospitalization, consistent with the survival and RMST analyses.

Clinical outcomes were based on symptom documentation in daily progress notes, which may introduce subjectivity and inter‑provider variability. Additionally, variability in patient availability at later time points reflects real‑world discharge practices and may limit outcome ascertainment.

Overall, these findings support limiting serum lipase testing to diagnostic purposes in pediatric AP and emphasize the importance of clinical assessment over biochemical monitoring. Future studies should focus on identifying more reliable clinical markers of disease progression, including standardized assessment of abdominal pain, as the absence of objective pain measures may contribute to continued reliance on laboratory trends [8].

This study is limited by its retrospective design, which restricts causal inference and is subject to documentation bias. The single‑center setting and relatively small sample size may limit generalizability. Decisions to trend serum lipase were based on individual clinician practice rather than a standardized protocol, introducing potential selection bias and confounding by clinical context. Variation in testing frequency precluded dose-response analyses, and some subgroup analyses were underpowered, particularly beyond 96 hours. Cost analyses were descriptive due to zero variance in the non‑trended group. Despite these limitations, the findings are consistent with existing literature and support guideline‑concordant management of pediatric AP.

## Conclusions

In this single‑center retrospective study, serial serum lipase measurement after diagnosis of pediatric AP was not associated with improved short‑term clinical outcomes. These findings support limiting lipase testing to diagnostic purposes and emphasize the importance of clinical assessment over biochemical monitoring.
